# Contrast volume and in-hospital outcomes of dialysis patients undergoing percutaneous coronary intervention

**DOI:** 10.1038/s41598-022-21815-y

**Published:** 2022-10-21

**Authors:** Toshiki Kuno, Yohei Numasawa, Satoshi Shoji, Ikuko Ueda, Masahiro Suzuki, Shigetaka Noma, Keiichi Fukuda, Shun Kohsaka

**Affiliations:** 1 Division of Cardiology, Montefiore Medical Center, Albert Einstein College of Medicine, 111 East 210Th St, New York, NY 10467-2401 USA; 2 Department of Cardiology, Japanese Red Cross Ashikaga Hospital, Ashikaga, Japan; 3 Department of Cardiology, Keio University School of Medicine, Tokyo, Japan; 4 Department of Cardiology, Saitama National Hospital, Wako, Japan; 5 Department of Cardiology, Saiseikai Utsunomiya Hospital, Utsunomiya, Japan

**Keywords:** Interventional cardiology, Kidney, Renal replacement therapy

## Abstract

Toxicity resulting from retained contrast media may cause adverse cardiovascular outcomes (e.g., heart failure and cardiogenic shock) for dialysis patients. However, the association between the administered contrast volume and outcomes of dialysis patients after percutaneous coronary intervention (PCI) has not been sufficiently investigated. We evaluated 953 consecutive dialysis patients (age, 67.9 ± 9.9 years; 30.1% with acute coronary syndrome) who underwent PCI between September 2008 and March 2019. Patients were divided into two groups: those with a contrast volume ≥ 200 ml and those with a contrast volume < 200 ml. The cutoff was 200 ml because 100 ml increment of contrast volume is known to raise the risk of acute kidney injury, and 200 ml is more than the average volume used at most PCI centers. The primary endpoint was a composite of in-hospital death, post-PCI cardiogenic shock and post-PCI heart failure. A multivariable logistic regression model and smooth spline curve were constructed to assess the association between contrast volume and the primary endpoint. The median contrast volume was 157 ml (interquartile range, 115–210 ml). The overall primary endpoint incidence was 6.8% (N = 65). A contrast volume ≥ 200 ml was associated with a higher risk of the primary endpoint (odds ratio 2.91; 95% confidence interval 1.42–6.05; P = 0.004). The smooth spline curve demonstrated a linear relationship between the contrast volume and primary endpoint. In conclusions, the contrast volume was associated with adverse in-hospital outcomes of dialysis patients undergoing PCI. Attention should be focused on the contrast volume used for dialysis patients undergoing PCI.

## Introduction

The contrast volume administered to patients undergoing percutaneous coronary intervention (PCI) is strongly associated with the risk of acute kidney injury (AKI)^1–4^. Clinical practice guidelines recommend minimizing the contrast volume to the lowest feasible level, especially for patients who are at high risk for AKI^3^. However, these recommendations are largely limited to non-dialysis patients. Several studies of dialysis patients have demonstrated that PCI operators focus little attention on the contrast volume because the contrast medium is cleared by subsequent dialysis^2,5,6^.

The association between the contrast volume and adverse outcomes other than AKI (e.g., heart failure and cardiogenic shock) for dialysis patients has been insufficiently investigated. A higher contrast volume is typically needed for PCI procedures performed for dialysis patients because of the higher incidence of complex coronary lesions; therefore, cardiac toxicity caused by higher concentrations of contrast media reaching the coronary arteries is a concern^7,8^. Additionally, acute expansion of plasma volume caused by osmotic effects may lead to heart failure.

We hypothesized that cardiovascular toxicity caused by the retained contrast media could result in adverse events, including new-onset cardiogenic shock and heart failure after PCI^7^. Using the contemporary multicenter all-comer PCI registry, we investigated the association between contrast volume and the risk of adverse in-hospital outcomes of dialysis patients undergoing PCI.

## Methods

### Database

This study was conducted as part of the Japan Cardiovascular Database-Keio Interhospital Cardiovascular Studies (JCD-KiCS) PCI registry, which is a multicenter, prospective registry including data of consecutive patients who underwent PCI between 2009 and 2017 at 15 institutions within the Tokyo metropolitan area. It primarily includes large tertiary care referral centers (≥ 200 beds; n = 13) and a few medium-sized satellite hospitals (< 200 beds; n = 2). The details of this registry have been published previously^2,9–13^. The participating hospitals were instructed to document and register patient data of consecutive hospital visits for PCI using an internet-based data collection system. Registered data were reviewed for completeness and internal consistency. Quality assurance of the data was achieved through automatic system validation, reporting of data completeness, and education and training of clinical research coordinators who were specifically trained to use the present PCI registry. The senior study coordinator (I.U.) and exclusive on-site auditing by the investigator (S.K.) ensured appropriate registration of each patient. All participants provided written informed consent. Before the launch of the JCD-KiCS registry, information regarding the objective of this registry was provided for clinical trial registration in the University Hospital Medical Information Network of Japan (UMIN000004736). The present study was approved by the institutional review board Committee of Keio University (Reference Number: 20080073), and was conducted in accordance with the principles of the Declaration of Helsinki. We also confirmed that all methods were performed in accordance with relevant guidelines and regulations.

### Definition of outcomes and variables

The clinical variables and outcomes of the JCD-KiCS were aligned with the data of the National Cardiovascular Data Registry CathPCI Registry version 4.1. Acute coronary syndrome (ACS) was defined as ST-segment elevation myocardial infarction (STEMI), non-STEMI, unstable angina. Stable coronary artery disease was defined as stable angina, previous myocardial infarction, and silent ischemia. The presence of heart failure was defined as documentation of heart failure by the attending physician, regardless of left ventricular ejection fraction. Multivessel disease was defined as two or more major coronary arteries with ≥ 75% stenosis. The estimated glomerular filtration rate was calculated using the Modification of Diet in Renal Disease Equation for Japanese Patients proposed by the Japanese Society of Nephrology^14–16^.

All major procedural complications (e.g., death, bleeding complications, and cardiac and cerebrovascular events) were defined by the clinical research coordinator. Initially, the procedural complications were reviewed by a trained clinical research coordinator under the supervision of the project coordinator and categorized as those in need of adjudication and those exempt from it. A separate member of the event committee reviewed the abstracted record. A second or third adjudicator was asked for assistance in the event of disagreement between the opinions of the project coordinator and the first adjudicator.

### Studied patients

Of the 24,162 consecutive PCI patients registered between September 2008 and March 2019, we selected 953 long-term dialysis patients and evaluated their in-hospital outcomes. Patients were divided into two groups: those who received a contrast volume ≥ 200 ml and those who received a contrast volume < 200 ml. The cutoff was set as 200 ml because 100 ml increment of contrast volume is known to be associated with the risk of AKI, and 200 ml is more than the average volume administered at most PCI centers; furthermore, a previous study showed that ≥ 200 ml of contrast volume was the precipitating factor for AKI^2,4,17^. Angiographical stenosis was defined as > 50% stenosis for left anterior descending artery, left circumflex artery and right coronary artery and ≥ 50% stenosis for left main.

The primary endpoint was defined as a composite of in-hospital death, post-PCI cardiogenic shock, and post-PCI heart failure. Post-PCI cardiogenic shock was defined as new-onset or acute recurrence of cardiogenic shock, a sustained (> 30 min) episode of systolic blood pressure < 90 mmHg, and/or cardiac index < 2.2 L/min/m^2^ determined to be secondary to cardiac dysfunction, and/or the requirement for parenteral inotropic or vasopressor agents or mechanical support (e.g., intra-aortic balloon pump, extracorporeal circulation, ventricular assist device) to maintain the blood pressure and cardiac index above the specified levels. Post-PCI heart failure was defined as new-onset or acute recurrence of heart failure that necessitated new or increased pharmacological therapy. A low ejection fraction without clinical evidence of heart failure was not considered heart failure.

The secondary endpoints were in-hospital mortality and PCI-related complications. PCI-related complications were defined as a composite endpoint that included severe flow-limiting coronary dissection/coronary perforation, myocardial infarction after PCI, post-PCI cardiogenic shock/heart failure, cerebral bleeding/stroke, and other bleeding complications defined as those requiring transfusion, prolonging the hospital stay, and/or reducing the hemoglobin level to < 3.0 g/dL^18^. When present, bleeding complications were classified as follows: puncture site bleeding, including external bleeding, or a hematoma > 10 cm for femoral sites, > 5 cm for brachial sites, or > 2 cm for radial access sites, retroperitoneal bleeding, gastrointestinal bleeding, genitourinary bleeding, or other bleeding types. This definition of bleeding-related complications was consistent with the Bleeding Academic Research Consortium definitions of grade 3A to grade 3C bleeds^19^.

### Statistical analyses

Continuous variables are presented as mean ± standard deviation or median (interquartile range), as appropriate, for data distribution. Categorical variables are expressed as percentages. The changes from baseline in continuous variables were evaluated using Student’s *t*-test or the Mann–Whitney *U* test. The χ^2^ or Fisher’s exact *t*-test was used to analyze categorical variables.

A multivariate logistic regression model was constructed to predict contrast volume ≥ 200 ml. Covariates were the followings; age, previous coronary bypass, culprit left main, culprit LAD, bifurcation, CTO, type C and use of rotational atherectomy.

A multivariate logistic regression model was also constructed to predict the incidence of the primary endpoint. Covariates were initially selected as the followings; age, baseline hemoglobin, heart failure at admission, cardiogenic shock, ACS, use of an intra-aortic balloon pump, three vessels disease, left main stenosis, contrast volume ≥ 200 ml. However, given the limited number of the primary endpoint, we generated a stepwise logistic regression model, which included age, baseline hemoglobin, cardiogenic shock, ACS, use of an intra-aortic balloon pump, three vessels disease, contrast volume ≥ 200 ml. Additionally, we checked the association between contrast volume and the risk-adjusted primary endpoint. The contrast volume was analyzed as a continuous variable using a smooth spline curve. During the subgroup analysis of patients who presented with ACS, we also performed a multivariable logistic regression analysis of the primary endpoint. Covariates were age, baseline hemoglobin, cardiogenic shock, STEMI, use of an intra-aortic balloon pump, three vessels disease and contrast volume ≥ 200 ml. All statistical calculations and analyses were performed using R 3.6.2 R Foundation for Statistical Computing (Vienna, Austria); p < 0.05 was considered statistically significant.

## Results

In this cohort, the mean age of the patients was 67.9 ± 9.9 years, and the baseline characteristics and in-hospital outcomes of patients administered a contrast volume ≥ 200 ml (N = 293; 30.7%) versus those who administered a contrast volume < 200 ml (N = 660; 69.3%) are shown in Tables 1 and 2. Patients administered a contrast volume ≥ 200 ml were younger and had significantly higher proportions of complex PCI, including bifurcation, chronic total occlusion, and type C lesions, and more frequently underwent rotational atherectomy and intravascular ultrasound (Table 1).

**Table 1:** Baseline characteristics of all patients; contrast volume < 200 ml versus contrast volume ≥ 200 ml.

	Contrast volume < 200 ml (N = 660)	Contrast volume ≥ 200 ml (N = 293)	P value
Age	68.5 ± 9.8	66.5 ± 10.2	0.003
Male	515 (78.0)	228 (77.8)	1.00
Baseline hemoglobin (g/dl)	10.7 [9.7, 11.7]	10.7 [9.8, 11.7]	0.70
Previous myocardial infarction	171 (25.9)	86 (29.4)	0.31
Previous heart failure	187 (28.3)	65 (22.2)	0.057
Diabetes mellitus	445 (67.8)	194 (67.1)	0.89
Cerebrovascular disease	104 (15.8)	55 (18.8)	0.29
Peripheral artery disease	164 (24.8)	72 (24.7)	1.00
Chronic lung disease	13 (2.0)	7 (2.4)	0.86
Hypertension	501 (75.9)	236 (80.5)	0.14
Dyslipidemia	317 (48.1)	144 (49.1)	0.82
Atrial fibrillation	71 (12.5)	19 (8.3)	0.12
Previous PCI	368 (55.8)	149 (50.9)	0.18
Previous coronary bypass	59 (8.9)	41 (14.0)	0.025
Heart failure on admission	92 (13.9)	42 (14.3)	0.95
Cardiogenic shock on admission	21 (3.2)	5 (1.7)	0.28
Cardiopulmonary arrest on admission	14 (2.1)	6 (2.0)	1.00
**Puncture site**			0.002
Femoral artery approach	579 (87.7)	269 (91.8)	
Radial artery approach	60 (9.1)	9 (3.1)	
Brachial artery approach	21 (3.2)	15 (5.1)	
Use of intra-aortic balloon pump	37 (5.6)	22 (7.5)	0.33
ST-elevation myocardial infarction	42 (6.5)	15 (5.2)	0.53
UA/NSTEMI	158 (24.6)	72 (25.1)	0.94
Acute coronary syndrome	200 (30.3)	87 (29.7)	0.91
Three vessels disease	153 (24.7)	71 (26.0)	0.745
**Angiographical stenosis**			
Left main	81 (12.6)	37 (13.0)	0.954
Left descending artery	453 (70.7)	202 (71.1)	0.95
Left circumflex	348 (54.3)	169 (59.7)	0.144
Right coronary artery	370 (58.2)	157 (56.3)	0.643
**Culprit vessel**			
Left main	29 (4.4)	25 (8.5)	0.016
Left descending artery	272 (41.2)	151 (51.5)	0.004
Left circumflex	165 (25.0)	82 (28.0)	0.373
Right coronary artery	238 (36.1)	82 (28.0)	0.018
Fluoroscopy time (min)	26.8 [16.5, 42.6]	45.1 [29.7, 72.4]	< 0.001
Contrast volume (ml)	130 [105, 160]	246 [218, 290]	< 0.001
Bifurcation lesion	163 (26.3)	114 (40.4)	< 0.001
Chronic total occlusion	42 (6.4)	36 (12.3)	0.003
Type C lesion	257 (41.1)	165 (58.9)	< 0.001
Use of intravascular ultrasound	521 (78.9)	256 (87.4)	0.003
Use of rotational atherectomy	59 (8.9)	71 (24.2)	< 0.001

PCI, percutaneous coronary intervention; UA/NSTEMI, unstable angina/non-ST-elevation myocardial infarction.

Data are presented as the mean ± standard deviation, number (%), and number [interquartile range].

**Table 2:** In-hospital mortality and complications.

	Contrast volume < 200 ml (N = 660), n (%)	Contrast volume ≥ 200 (N = 293), n (%)	P value
Primary endpoint	40 (6.1)	25 (8.5)	0.21
In-hospital mortality	28 (4.3)	15 (5.1)	0.67
All complications	49 (7.5)	41 (14.3)	0.002
Coronary dissection	1 (0.2)	4 (1.4)	0.056
Coronary perforation	4 (0.6)	5 (1.7)	0.21
Myocardial infarction	4 (0.6)	7 (2.4)	0.04
Cardiogenic shock	18 (2.7)	12 (4.1)	0.36
Heart failure	8 (1.2)	7 (2.4)	0.29
Cerebral infarction	2 (0.3)	2 (0.7)	0.77
Intracranial hemorrhage	0 (0.0)	1 (0.3)	0.68
Cardiac tamponade	1 (0.2)	0 (0.0)	1.00
Transfusion	26 (3.9)	20 (6.8)	0.079
Bleeding (all types)	22 (3.3)	16 (5.5)	0.17
Puncture site bleeding	9 (1.4)	2 (0.7)	0.56
Puncture site hematoma	4 (0.6)	4 (1.4)	0.42
Peritoneal bleeding	0 (0.0)	1 (0.3)	0.68
Gastrointestinal bleeding	3 (0.5)	3 (1.0)	0.56
Genitourinary bleeding	1 (0.2)	0 (0.0)	1.00
Other bleeding	8 (1.2)	8 (2.7)	0.16

The overall incidence of the primary endpoint was 6.8% (N = 65). The crude primary endpoints were similar for patients who did and did not receive a contrast volume ≥ 200 ml (Table 2). Additionally, we did a multivariable logistic regression model for the predictor of a contrast volume ≥ 200 ml, showing younger age, culprit of left descending artery, culprit of left main, bifurcation lesion, chronic total occlusion, type C lesion and use of rotational atherectomy were the predictors of a contrast volume ≥ 200 ml (Table 3).

**Table 3:** Multivariable logistic regression model of the factor for contrast ≥ 200 ml.

	Odds ratio	Confidential interval	P value
Age	0.98	0.96–0.99	0.004
Previous coronary bypass	1.51	0.93–2.45	0.093
Culprit left main	1.55	0.82–2.91	0.176
Culprit left descending artery	1.37	1.01–1.87	0.046
Bifurcation lesion	1.56	1.11–2.18	0.009
Chronic total occlusion	2.08	1.21–3.56	0.008
Type C lesion	1.39	1.001–1.92	0.048
Use of rotational atherectomy	2.92	1.95–4.40	< 0.001

**Figure 1:**
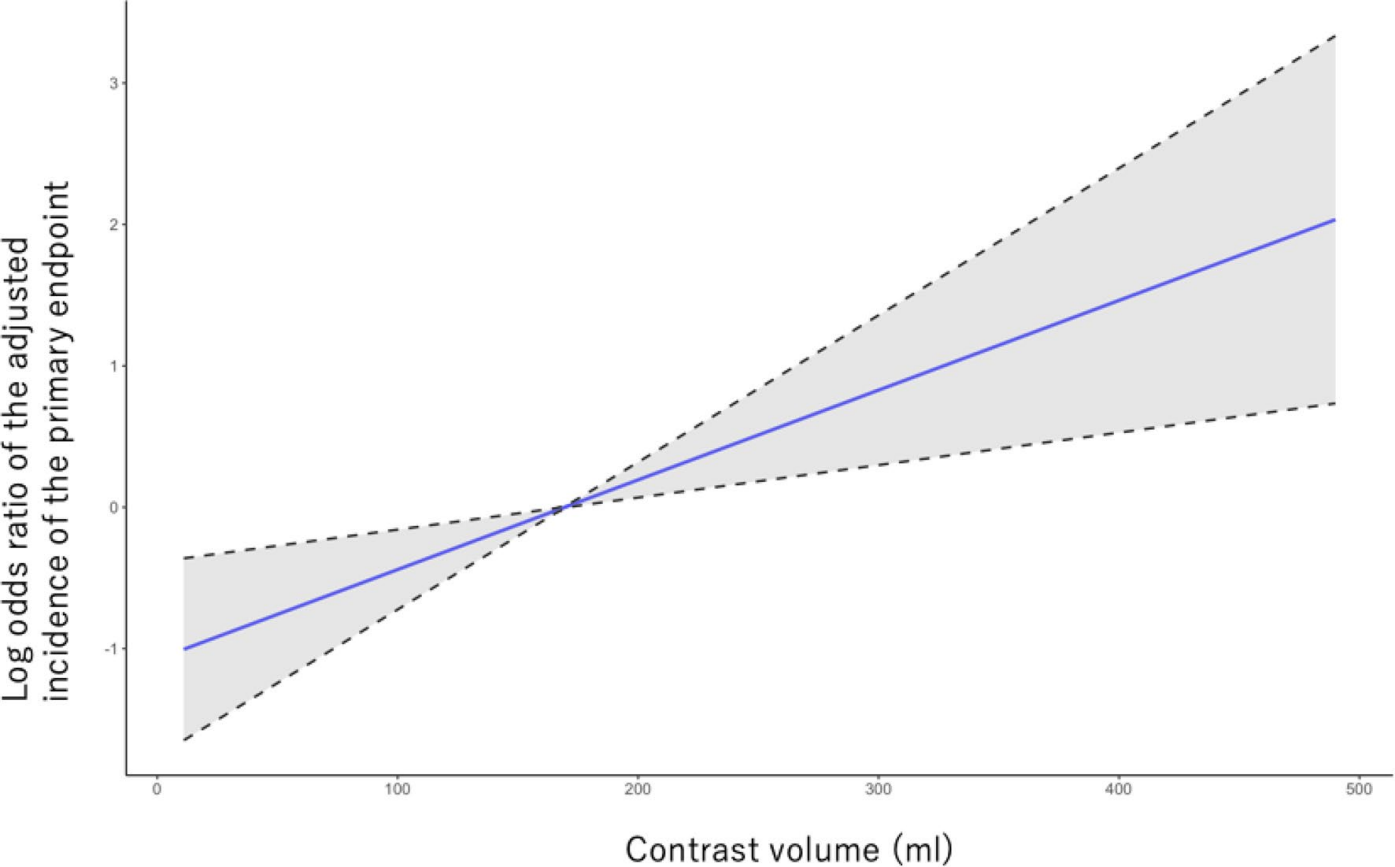
Smooth spline showing the association between contrast volume and the primary endpoint. The y axis shows the log odds ratio of the adjusted incidence of the primary endpoint. The x axis shows the contrast volume. The gray area shows the confidence interval.

Table 4 shows the patients’ characteristics of those with the primary endpoint and those without. The multivariable logistic regression model demonstrated that the use of a contrast volume ≥ 200 ml was an independent predictor of the incidence of the primary endpoint (odds ratio [OR] 2.91; 95% confidence interval [CI] 1.42–6.05; P = 0.004), as well as for in-hospital death (OR 2.78; 95% CI 1.16–6.81; P = 0.022). Other predictors of the primary endpoint are shown in Table 5. The adjusted smooth spline curve demonstrated a linear relationship between the contrast volume and the primary endpoint (Fig. 1).

**Table 4:** Baseline characteristics of all patients; patients with primary endpoint versus those without.

	Patients without the incidence of primary endpoint (N = 888), n (%)	Patients with the incidence of primary endpoint (N = 65), n (%)	P value
Age	67.65 (9.96)	70.89 (9.40)	0.011
Male	693 (78.0)	50 (76.9)	0.956
Baseline hemoglobin (g/dl)	10.80 [9.80, 11.80]	10.00 [8.90, 10.70]	< 0.001
Previous myocardial infarction	235 (26.5)	22 (33.8)	0.25
Previous heart failure	223 (25.1)	29 (44.6)	0.001
Diabetes mellitus	592 (67.1)	47 (74.6)	0.277
Cerebrovascular disease	147 (16.6)	12 (18.5)	0.821
Peripheral artery disease	220 (24.8)	16 (24.6)	1.00
Chronic lung disease	17 (1.9)	3 (4.6)	0.309
Hypertension	687 (77.4)	50 (76.9)	1.00
Dyslipidemia	422 (47.6)	39 (60.0)	0.071
**Atrial fibrillation**			
Previous PCI	481 (54.2)	36 (55.4)	0.951
Previous coronary bypass	91 (10.2)	9 (13.8)	0.481
Heart failure on admission	111 (12.5)	23 (35.4)	< 0.001
Cardiogenic shock on admission	12 (1.4)	14 (21.5)	< 0.001
Cardiopulmonary arrest on admission	10 (1.1)	10 (15.4)	< 0.001
**Puncture site**			0.426
Femoral artery approach	790 (89.0)	58 (89.2)	
Radial artery approach	66 (7.4)	3 (4.6)	
Brachial artery approach	32 (3.6)	4 (6.2)	
Use of intra-aortic balloon pump	31 (3.5)	28 (43.1)	< 0.001
ST-elevation myocardial infarction	45 (5.2)	12 (18.5)	< 0.001
UA/NSTEMI	205 (23.7)	25 (38.5)	0.012
Acute coronary syndrome	250 (28.2)	37 (56.9)	< 0.001
Three vessels disease	200 (24.1)	24 (38.7)	0.016
**Angiographical stenosis**			
Left main	99 (11.5)	19 (29.2)	< 0.001
Left descending artery	605 (70.3)	50 (76.9)	0.326
Left circumflex	474 (55.1)	43 (68.3)	0.057
Right coronary artery	484 (56.9)	43 (67.2)	0.139
**Culprit vessel**			
Left main	44 (5.0)	10 (15.4)	0.001
Left descending artery	392 (44.1)	31 (47.7)	0.67
Left circumflex	226 (25.5)	21 (32.3)	0.284
Right coronary artery	298 (33.6)	22 (33.8)	1.00
Fluoroscopy time (min)	31.20 [19.95, 51.15]	29.20 [21.52, 63.35]	0.601
Contrast volume (ml)	157.50 [115.00, 208.00]	157.00 [115.00, 240.00]	0.344
Contrast volume ≥ 200 ml	268 (30.2)	25 (38.5)	0.209
Bifurcation lesion	253 (30.2)	24 (38.1)	0.242
Chronic total occlusion	74 (8.3)	4 (6.2)	0.701
Type C lesion	385 (45.8)	37 (56.9)	0.11
Use of intravascular ultrasound	726 (81.8)	51 (78.5)	0.62
Use of rotational atherectomy	119 (13.4)	11 (16.9)	0.541

*PCI* percutaneous coronary intervention, *UA/NSTEMI* unstable angina/non-ST-elevation myocardial infarction.

Data are presented as the mean ± standard deviation, number (%), and number [interquartile range].

**Table 5:** Multivariable logistic regression model of the primary endpoint.

	Odds ratio	Confidential interval	P value
Age	1.05	1.01–1.09	0.007
Baseline hemoglobin	0.52	0.40–0.66	< 0.001
Cardiogenic shock at presentation	3.95	1.17–13.3	0.026
Acute coronary syndrome	1.90	0.96–3.85	0.069
Use of intra-aortic balloon pump	18.4	7.76–45.1	< 0.001
Three vessels disease	1.79	0.85–3.71	0.116
Contrast volume ≥ 200 ml	2.91	1.42–6.05	0.004

The subgroup analysis of patients with ACS (N = 287) demonstrated similar findings. The use of ≥ 200 ml of contrast media was also an independent predictor of the incidence of the primary endpoint (OR 4.32; 95% CI 1.71–11.4; P = 0.002), as well as in-hospital death (OR 3.71; 95% CI 1.29–11.1; P = 0.016).

## Discussion

During this study, we found that the administration of ≥ 200 ml of contrast media was an independent predictor of the incidence of the primary endpoint (the composite in-hospital death, post-PCI cardiogenic shock, and post-PCI heart failure). Furthermore, the smooth spline curve revealed a linear relationship between the contrast volume and primary endpoint.

AKI is common in patients undergoing PCI and is associated with increased risks of short-term and long-term mortality^20,21^. Therefore, PCI operators focus attention on the contrast volume administered to non-dialysis patients who undergo PCI^4^. However, in current practice, they do not focus attention on the contrast volume administered to dialysis patients because they are already on dialysis and there is no perceived risk of AKI. Nevertheless, our data demonstrated that the contrast volume was associated with adverse in-hospital outcomes; therefore, PCI operators should focus attention on the amount of contrast media administered.

Contrast media reaching the coronary arteries in high concentrations can affect cardiac output^7,8^, and acute expansion of the plasma volume by osmotic effects is considered to affect hemodynamics, resulting in acute pulmonary edema with an increase in systemic blood pressure because dialysis patients have impaired excretion of contrast media^7^. Therefore, toxic cardiovascular effects caused by retained contrast media can result in cardiogenic shock, heart failure, and in-hospital death after PCI^7^. This is a novel finding because no studies have investigated the association of contrast volume and in-hospital outcomes of dialysis patients. Although contrast media can be dialyzable^22,23^, our study could not investigate the effect of dialysis after PCI because we did not have sufficient information, which was a limitation of our study. Further studies are needed to investigate the utility of dialysis immediately after PCI for dialysis patients.

We constructed a fully adjusted smooth spline curve that illustrated that the contrast volume was linearly associated with in-hospital outcomes of dialysis patients who underwent PCI. We set the cutoff of the contrast volume to 200 ml in the multivariable logistic regression model. Our findings that PCI operators should minimize the contrast volume to decrease the risk of adverse in-hospital outcomes for these patients could be applied in clinical practice.

Our study had several limitations. First, we selected our patient cohort from a prospective observational study that was not designed to enable a focused investigation of the association between contrast volume and in-hospital outcomes of dialysis patients. Second, we excluded dialysis patients who did not have any contrast volume information. Third, we did not have information about the timing of dialysis before and after PCI, which may have affected the events of cardiogenic shock or heart failure after PCI. However, we demonstrated that the amount of contrast media was associated with worse in-hospital outcomes for ACS patients who relatively did not have time to undergo dialysis before PCI because of the urgency to undergo PCI compared to patients who underwent elective PCI. This finding also demonstrates the robustness of the data. Fourth, we did not have information about the types of contrast media, which would have affected the outcomes because lower-osmolarity contrast media may not require immediate dialysis to avoid hemodynamic effects^24^. Nonetheless, the data were mainly derived from the use of less than 100 ml of contrast media, suggesting that our data showing the risk of using more than 200 ml of contrast media is meaningful. Fifth, we did not have information of time course of events to assess the association of the contrast volume and the primary endpoint. Finally, we showed the association of the contrast volume and adverse outcomes after PCI. However, we could not conclude whether the contrast volume affected outcomes or whether patients who needed more contrast volume had worse outcomes. Further studies investigating liberal versus restrictive contrast use are needed to confirm our findings.

In conclusion, contrast volume was associated with the risk of adverse in-hospital outcomes among dialysis patients undergoing PCI. Attention should be focused on the contrast volume used for dialysis patients undergoing PCI.

## Data Availability

The data that support the findings of this study are available from JCD-KiCS but restrictions apply to the availability of these data, which were used under license for the current study, and so are not publicly available. Data are however available from the authors upon reasonable request and with permission of JCD-KiCS to the corresponding author.

## Abbreviations

ACS: Acute coronary syndrome
AKI: Acute kidney injury
JCD-KiCS: Japan Cardiovascular Database-Keio Interhospital Cardiovascular Studies
PCI: Percutaneous coronary intervention
STEMI: ST-segment elevation myocardial infarction
